# Microvascular Networks From Endothelial Cells and Mesenchymal Stromal Cells From Adipose Tissue and Bone Marrow: A Comparison

**DOI:** 10.3389/fbioe.2018.00156

**Published:** 2018-10-25

**Authors:** Karoline Pill, Johanna Melke, Severin Mühleder, Marianne Pultar, Sabrina Rohringer, Eleni Priglinger, Heinz R. Redl, Sandra Hofmann, Wolfgang Holnthoner

**Affiliations:** ^1^Ludwig Boltzmann Institute for Experimental and Clinical Traumatology, Vienna, Austria; ^2^Austrian Cluster for Tissue Regeneration, Vienna, Austria; ^3^Orthopaedic Biomechanics, Department of Biomedical Engineering, Eindhoven University of Technology, Eindhoven, Netherlands; ^4^Institute for Complex Molecular Systems, Eindhoven University of Technology, Eindhoven, Netherlands; ^5^Department of Biomedical Research, Medical University of Vienna, Vienna, Austria

**Keywords:** tissue engineering, endothelial cells, mesenchymal stromal cells, adipose-derived, bone marrow-derived

## Abstract

A promising approach to overcome hypoxic conditions in tissue engineered constructs is to use the potential of endothelial cells (EC) to form networks *in vitro* when co-cultured with a supporting cell type in a 3D environment. Adipose tissue-derived stromal cells (ASC) as well as bone marrow-derived stromal cells (BMSC) have been shown to support vessel formation of EC *in vitro*, but only very few studies compared the angiogenic potential of both cell types using the same model. Here, we aimed at investigating the ability of ASC and BMSC to induce network formation of EC in a co-culture model in fibrin. While vascular structures of BMSC and EC remained stable over the course of 3 weeks, ASC-EC co-cultures developed more junctions and higher network density within the same time frame. Both co-cultures showed positive staining for neural glial antigen 2 (NG2) and basal lamina proteins. This indicates that vessels matured and were surrounded by perivascular cells as well as matrix molecules involved in stabilization. Gene expression analysis revealed a significant increase of vascular endothelial growth factor (VEGF) expression in ASC-EC co-culture compared to BMSC-EC co-culture. These observations were donor-independent and highlight the importance of organotypic cell sources for vascular tissue engineering.

## Introduction

One of the major limitations of tissue engineered constructs is their need of a vascular system once they are implanted *in vivo*, as the diffusion limit of oxygen is smaller than 200 μm (Jain et al., 2005). A promising approach to overcome this shortage is to use the capacity of endothelial cells (EC) to form vessel-like structures if cultured with a supporting cell type like adipose tissue-derived stromal cells (ASC), bone marrow-derived stromal cells (BMSC) or fibroblasts in a three dimensional (3D) environment *in vitro*. In previous studies, EC started to interconnect with each other to form tube structures with lumen that were able to anastomose with the host vascular network upon implantation *in vivo* (Melero-Martin et al., 2008; Reinisch et al., 2009; Traktuev et al., 2009; Unger et al., 2010; Verseijden et al., 2010a, 2012; Pill et al., 2015; Tiruvannamalai Annamalai et al., 2016). Also endothelial colony forming cells (ECFC) supported by BMSC in a 3D matrix of different hydrogels showed network formation 7 days after injection into immunodeficient mice (Allen et al., 2011). Similar structures were found in ECFC-ASC co-cultures supported by a collagen-fibronectin matrix 2 weeks after implantation (Traktuev et al., 2009). The vascular structures matured over time by surrounding themselves with perivascular cells and forming a physiological basal lamina *in vitro* (Pill et al., 2015; Tiruvannamalai Annamalai et al., 2016).

Although mesenchymal stromal cells (MSC) of different sources share similar characteristics, they have been shown to induce vascular tube formation via distinct molecular interactions (Dominici et al., 2006; Pill et al., 2015). While angiogenic factor release (Rohringer et al., 2014; Katagiri et al., 2017) and induction of network formation (Pill et al., 2015) seem to be similar in ASC and BMSC, distinct differences were reported regarding matrix degradation (Kachgal and Putnam, 2011). The direct comparison between these two supporting cell types is difficult as only few groups investigated differences in the same experimental setting using both cell sources. To systematically asses the differences between these two cell sources as supporters of EC, a direct comparison of both within the same system seems crucial. In this study we aimed to identify similarities and differences of ASC- and BMSC-induced vascular network formation in the same 3D co-culture setting *in vitro* to investigate which cell type might be favorable for distinct vascularization approaches.

## Materials and methods

### Cell isolation and culture

The isolation of EC as well as ASC was approved by the ethics committee of Upper Austria (ethics vote #200) and performed after patients gave written informed consent. Research was performed in accordance with relevant guidelines and regulations. Human umbilical vein endothelial cells (HUVEC, from here on termed EC) were isolated as described elsewhere (Petzelbauer et al., 1993). Briefly, clamped umbilical cords were cut from the placenta and cleaned from superficial blood. Clamps were removed by cutting. 10 cm of a 150 cm Perfusor® line were cut and connected to a 20 ml syringe filled with PBS. The tubing was inserted into the vein, fixed with a clamp and the vein was washed with PBS to remove clotted blood. Then, the other end of the umbilical cord was clamped, the vein was filled with 1x trypsin and sealed by putting the second clamp onto the cord. The entire umbilical cord was then incubated in pre-warmed PBS at 37°C for 15 min. The clamp was removed on one site and the cell suspension was massaged out of the vein into a sterile tube. Subsequently, the cord was washed with 1x PBS and the wash-out was collected. After addition of 10 ml Endothelial Growth Medium-2 (EGM-2, Lonza) the entire cell suspension was then centrifuged and the pellet was resuspended in EGM-2 growth medium supplemented with additional fetal calf serum (FCS, Sigma-Aldrich Cat No: F9665-500ML) to a final concentration of 5%. HUVEC were used from single donors at passage 5 for all experiments. ASC as well as BMSC were differentiated towards an adipogenic, osteogenic and chondrogenic phenotype to confirm pluripotency (Supplementary Methods). ASC were isolated from liposuction material as previously described (Wolbank et al., 2007; Priglinger et al., 2017) and cultured in EGM-2 supplemented with additional FCS to a final concentration of 5% and used from single donors at passage 5 for all experiments. BMSC were isolated and expanded from whole bone marrow obtained from Lonza by cell adhesion to tissue culture plastic as previously described (Hofmann et al., 2007) and also used from single donors at passage 5 for all experiments. They were cultured in high glucose Dulbeccos's modified Eagle's medium (DMEM, Sigma-Aldrich) supplemented with 10% FCS (Bovogen Biologicals), 1% Penicillin/Streptomycin, 1% non-essential amino acids (Sigma-Aldrich), and 1 ng/ml basic fibroblast growth factor (bFGF, Peprotech).

### Retroviral infection

HUVEC were retrovirally infected with yellow fluorescent protein (YFP), green fluorescent protein (GFP) or red fluorescent protein (mCherry) to document network-formation over time as previously described (Knezevic et al., 2017). Briefly, cDNA for eGFP and mCherry (Addgene) and eYFP-HIS (ThermoFisher) were subcloned into the pBMN backbone (pBMN-LacZ, Addgene). Virus particles were generated by transfecting Phoenix Ampho cells [a kind gift from Regina Grillari (University of Natural Resources and Life Sciences, Vienna, Austria)] and the virus-containing supernatant was used to infect the target cells.

### Fibrin matrix co-culture and network quantification

EC co-cultures with ASC or BMSC in fibrin matrices were prepared as previously described (Rohringer et al., 2014). In brief, 10^5^ YFP- or GFP-EC were embedded together with either 10^5^ ASC or 10^5^ BMSC in 200 μl fibrin matrices (TISSEEL®, Baxter) with a final concentration of 2.5 mg/ml fibrinogen and 0.2 IU/ml thrombin. 10^5^ EC cultured in fibrin without any other cell type were termed “EC only” and served as control. Two different donors of each cell type (ASC, BMSC, and EC, six different donors in total) were investigated in different combinations of four biological replicates. Each sample was generated as technical duplicate (two matrices). All matrices were cultured in EGM-2, 5% FCS. Two representative, fluorescent images of approximately the same area of each matrix were taken on day 1, day 7, day 14, and day 21 of culture on an epifluorescence microscope (Zeiss Observer A1, Zeiss) for further analysis, leading to 16 different values for each data set. Images taken from co-cultures at day 7, day 14, and day 21 were used to quantify network parameters number of junctions, number of tubules, mean tubule length, and total tubule length. All images were randomized and processed first with Photoshop (CS5, Adobe) and subsequently with Angiosys software (TCS Cellworks) (Charwat et al., 2015; Knezevic et al., 2017). For lumen investigations, z-stacks were recorded after 15 days of co-culture using a confocal microscope (Zeiss LSM510).

### Immunostaining (NG2, collagen type IV, laminin, and perlecan)

The protocol for immunostaining against NG2 was adapted from Eglinger et al. (2017) and performed on four different biological replicates (each as technical duplicate). After 21 days, fibrin matrices containing cells were washed three times with PBS for 5 min at room temperature (RT), fixed with 4% paraformaldehyde (in PBS, Sigma-Aldrich) for 2 h, washed again three times with PBS and subsequently incubated with blocking solution (PBS containing 3% Triton X-100 [Sigma-Aldrich] and 1% bovine serum albumin [Sigma-Aldrich]) overnight at 37°C. The next day, anti-NG2 antibody (anti-human NG2/MCSP PE-conjugated, monoclonal mouse, catalog# FAB2585P, R&D Systems) diluted 1:200 in blocking solution was added and incubated for at least 70 h at 37°C. The supernatant was then discarded and washing solution (PBS containing 3% Triton X-100) added. Samples were washed for at least 40 h at 37°C and the washing solution was exchanged three times during this period. Secondary antibody (anti-mouse Alexa Fluor 594, polyclonal goat, catalog# A11005, Invitrogen) was diluted 1:500 in washing solution, added to the samples and incubated for at least 40 h at 37°C. After washing again three times with washing buffer, samples were imaged using fluorescence microscopy.

Immunostainings against collagen type IV, laminin and perlecan were performed on co-cultures of mCherry-EC and ASC and mCherry-EC and BMSC after 1 week of culture, as these proteins were reported to be deposited after this time span in distinct co-cultures (Ganesan et al., 2017). Two different ASC and EC donors and one BMSC donor were investigated in two biological replicates. Matrices were washed three times with PBS (at least once for 15 min). Samples were subsequently fixed with 4% paraformaldehyde (in PBS, Sigma-Aldrich) overnight at 4°C. Cells were washed again three times with PBS (at least once for 15 min) and blocking step was performed by adding PBS containing 1% bovine serum albumin for 2 h at 4°C. Afterwards, the primary antibodies were added (anti-human laminin, polyclonal rabbit, catalog# ab11575 or anti-human collagen IV, polyclonal rabbit, catalog# ab6586 or anti-human heparan sulfate proteoglycan 2, monoclonal mouse, catalog# ab23418 all three from Abcam), diluted 1:100 in PBS with 1% bovine serum albumin overnight at 4°C. Samples were washed three times with PBS prior to adding the secondary antibody (anti-rabbit Alexa Fluor 488, polyclonal goat, catalog# A11034 or anti-mouse Alexa Fluor 488, polyclonal goat, catalog# A11029 both from Invitrogen) diluted 1:100 in PBS containing 1% bovine serum albumin overnight at 4°C. Samples were washed three times with PBS and imaged using fluorescence microscopy.

### RNA isolation and qRT-PCR

Fibrin matrices containing cells were harvested on day 1, day 7, day 14, and day 21, shock-frozen in liquid nitrogen and subsequently homogenized with a Dstroy S-15 stick (Biozym). For RNA isolation the samples were processed with peqGOLD TriFast™ (Peqlab). RNA concentration was measured with a spectrophotometer (Nanodrop OneC, Thermo Fisher Scientific). Approximately 2 μg RNA of each sample were treated with DNase I (Promega) at 37°C for 30 min. Subsequently, complementary DNA (cDNA) was synthesized using an EasyScript™ cDNA Synthesis Kit (abmGood). For quantitative real time PCR, 20 ng cDNA, 400 nM primer-mix (forward and reverse, Microsynth, Table 1, ANGPT1 and ANGPT2 from (Stahl et al., 2004)) and 10 μl PerfeCTa® SYBR® Green SuperMix (Quanta Biosciences) were mixed with ddH_2_O to a final volume of 20 μl for each well. Combinations of two different ASC, BMSC and EC donors were investigated in four biological replicates (each in technical duplicate). All samples were measured in triplicates using a real time detection system (Bio-Rad CFX96 Touch™ Real-Time PCR Detection System). The denaturing step was performed at 95°C for 180 s, then 50 cycles of 95°C for 10 s, annealing temperature for 30 s (Table 1) and 72°C elongation temperature for 10 s were performed. Values are normalized to *GAPDH* and day 1.

**Table 1:** Primers and annealing temperatures (T_a_) used for quantitative real time PCR.

**Gene**	**GenBank Accession number**	**Protein function**	**Forward**	**Reverse**	**T_a_**
***GAPDH*** Glyceraldehyde 3-phosphate dehydrogenase	NM_002046.3	Basic enzyme	5′ GTC AGC CGC ATC TTC TTT TG 3′	5′ CCC AAT ACG ACC AAA TCC G 3′	55°C
***VEGFA*** Vascular endothelial growth factor A	NM_001317010.1	Main angiogenic growth factor	5′ AGG CAG CTT GAG TTA AAC 3′	5′ CTG GAT TAA GGA CTG TTC TG 3′	55°C
***PECAM1*** Platelet endothelial cell adhesion molecule 1	NM_000442.4	Homotypic EC-EC connection	5′ CTT GGA GTC CTG CTG ACC 3′	5′ AGA GGT GGT GCT GAC ATC 3′	55°C
***TEK*** Tyrosine endothelial kinase	NM_000459.4	Angiopoietin receptor	5′ GGA GAG GCA ATC AGG ATA 3′	5′ GAG GCA GGT GTA CTT CTA 3′	55°C
***CDH5*** Cadherin 5	NM_001795.4	Transmembrane adhesion molecule	5′ CCA GAT GCA CAT TGA TGA A 3′	5′ TCT CCT TTG AGC AGG TAC 3′	55°C
***KDR*** Kinase insert domain-containing receptor	NM_002253.2	VEGFA receptor	5′ TGT CGT TGT AGG GTA TAG G 3′	5′ TCT CCA ACA GAT AGT TCA ATT C 3′	50°C
***PDGFB*** Platelet-derived growth factor B-chain	NM_002608.3	Growth factor	5′ CAA GCA CCG GAA ATT CAA 3′	5′ GGC AAT ACA GCA AAT ACC A 3′	50°C
***VWF*** Von Willebrand factor	NM_000552.4	Endothelial derived thrombocyte adhesion molecule	5′ CTC GAT TAT TGG GGA CTT C 3′	5′ GAC AGC AGG ACT TGA AAG 3′	59°C
***ANGPT1*** Angiopoietin 1	NM_001146.4	Blood vessel stabilization and destabilization	5′ TCGCTGCCATTCTGACTCAC 3′	5′ CCGGTTATATCTTCTCCCACTGTT 3′	60°C
***ANGPT2*** Angiopoietin 2	NM_001147.2	Blood vessel stabilization and destabilization	5′ TCCTCCTGCCAGAGATGGAC 3′	5′ TGCACAGCATTGGACACGTA 3′	60°C

### Statistics

Statistical analyses were performed using GraphPad Prism Version 5 (GraphPad). For comparing vascular parameters obtained through network quantification within a time course (day 7, day 14, day 21) a Friedman test was performed with Dunns *post*-*hoc*-test. Vascular parameters of networks derived from co-cultures of EC and ASC were compared with co-cultures of EC and BMSC at each time point using two-tailed Mann–Whitney test. For real time PCR data, the time course of each target was analyzed using Kruskal–Wallis test with Dunns *post-hoc*-test. To compare network parameters and real time PCR data of EC and ASC co-cultures with EC and BMSC co-cultures for each separate time point, a Mann–Whitney test was performed. Values were considered significant when *p* < 0.05.

## Results

### ASC as well as BMSC induce network formation in EC which remain stable over 3 weeks of culture in a 3D fibrin matrix

**Figure 1:**
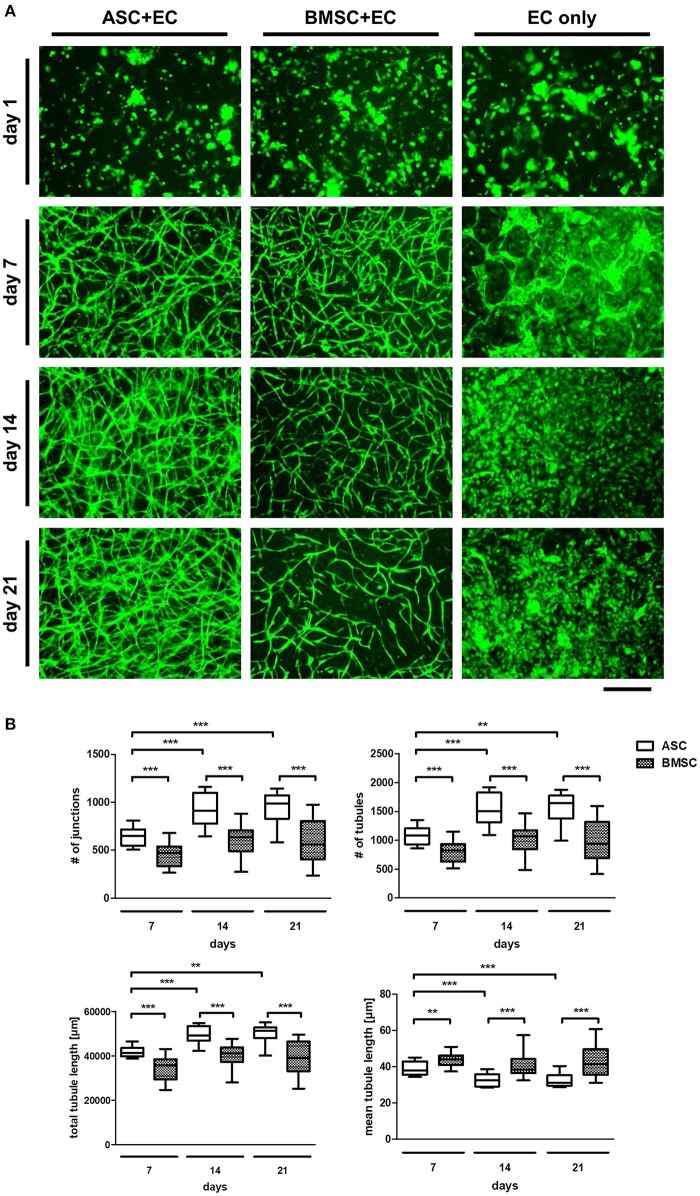
ASC induce a denser network of tubular structures in EC than BMSC in a 3D fibrin matrix, while in both groups the networks remain stable over 3 weeks of culture. **(A)** Representative fluorescent images from all three groups (ASC + EC, BMSC + EC, and EC only) at all-time points. EC are depicted in green. **(B)** Network parameters number of junctions, number of tubules and total tubule length were significantly higher and mean tubule length significantly lower in ASC + EC compared to BMSC + EC co-cultures at all time-points. All parameters changed significantly in ASC + EC between day 7 and day14/day21. *n* = 16 from four biological replicates (each in technical duplicate), comparing two different donors of each cell type, **p < 0.01, ***p < 0.001. Scale bar: 300 μm.

To confirm the pluripotency of the used progenitor cells, the differentiation potential of ASC and BMSC as well as cell surface marker expression were investigated (Figure S1). MSC populations were able to differentiate toward an adipogenic, osteogenic, and chondrogenic phenotype and were positive for CD73, CD90, and CD105 and negative for CD31, CD14, and CD45. Consequently, the ability of MSC to induce network formation in EC over a time period of 21 days using fluorescently labeled EC co-cultured with non-fluorescent ASC or BMSC in a 3D fibrin matrix was investigated (Figure 1A). While all three groups (ASC+EC, BMSC+EC, and EC only) showed a homogenous distribution of green fluorescent EC on day 1, only co-cultures started to form networks after 4 days of culture (Rohringer et al., 2014) which were then quantified after 7 days of culture. Tubular structures were comparable in density and morphology. However, from day 14 onwards, ASC+EC co-cultures formed denser networks than BMSC+EC co-cultures. Both co-cultures showed hollow structures indicating lumen formation of vascular structures formed by EC after 15 days (Figure S2). Tubular structures formed in both co-cultures remained stable over the duration of 3 weeks. EC without any contact to supporting cells did not form tubular structures within the fibrin matrix.

Quantifying network parameters revealed a significantly higher number of junctions, number of tubules and a longer total tubule length and a significantly lower mean tubule length when comparing ASC+EC co-cultures with BMSC+EC co-cultures at all-time points (day 7, day 14, and day 21, Figure 1B). This indicates that ASC led to more and shorter tubules and therefore higher vascular density. Furthermore, none of the parameters changed significantly over time in BMSC+EC co-cultures in contrast to ASC+EC co-cultures, where all parameters either significantly increased (number of junctions, number of tubules, total tubule length) or decreased (mean tubule length) between day 7 and day 21.

### **ASC as well as BMSC** differentiate toward a pericyte phenotype in close proximity to vascular networks

**Figure 2:**
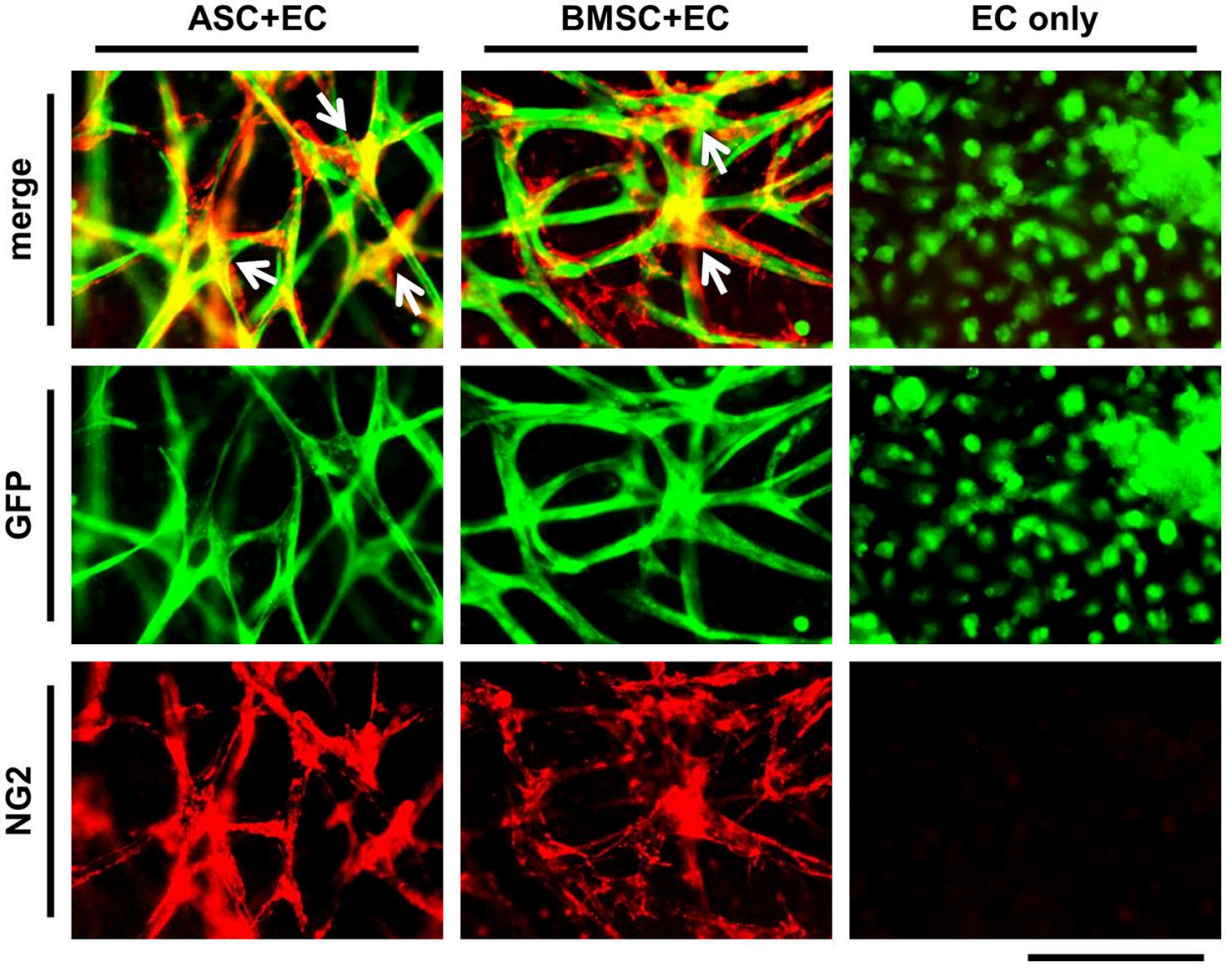
ASC as well as BMSC differentiate toward a pericyte phenotype in close proximity to vessel-like structures. EC networks (green) are surrounded by NG2 expressing cells (red) in both co-cultures, while a stronger signal could be observed at the junctions (white arrows) after 3 weeks of culture. Scale bar: 150 μm.

To show vessel maturation at the end of the culture period of 3 weeks, the differentiation of MSC toward a perivascular cell within co-culture was investigated. ASC+EC co-cultures or BMSC+EC co-cultures were fixed and stained against NG2 (Figure 2, EC are depicted in green, NG2 in red). We observed that nearly the entire networks in both co-cultures were covered with cells expressing NG2. Positive cells were localized to a higher extent at the junctions of vessels. There was no difference in localization comparing both co-cultures. EC only cultures lacked NG2.

### Basal lamina components are deposited in ASC+EC as well as BMSC+EC co-cultures

**Figure 3:**
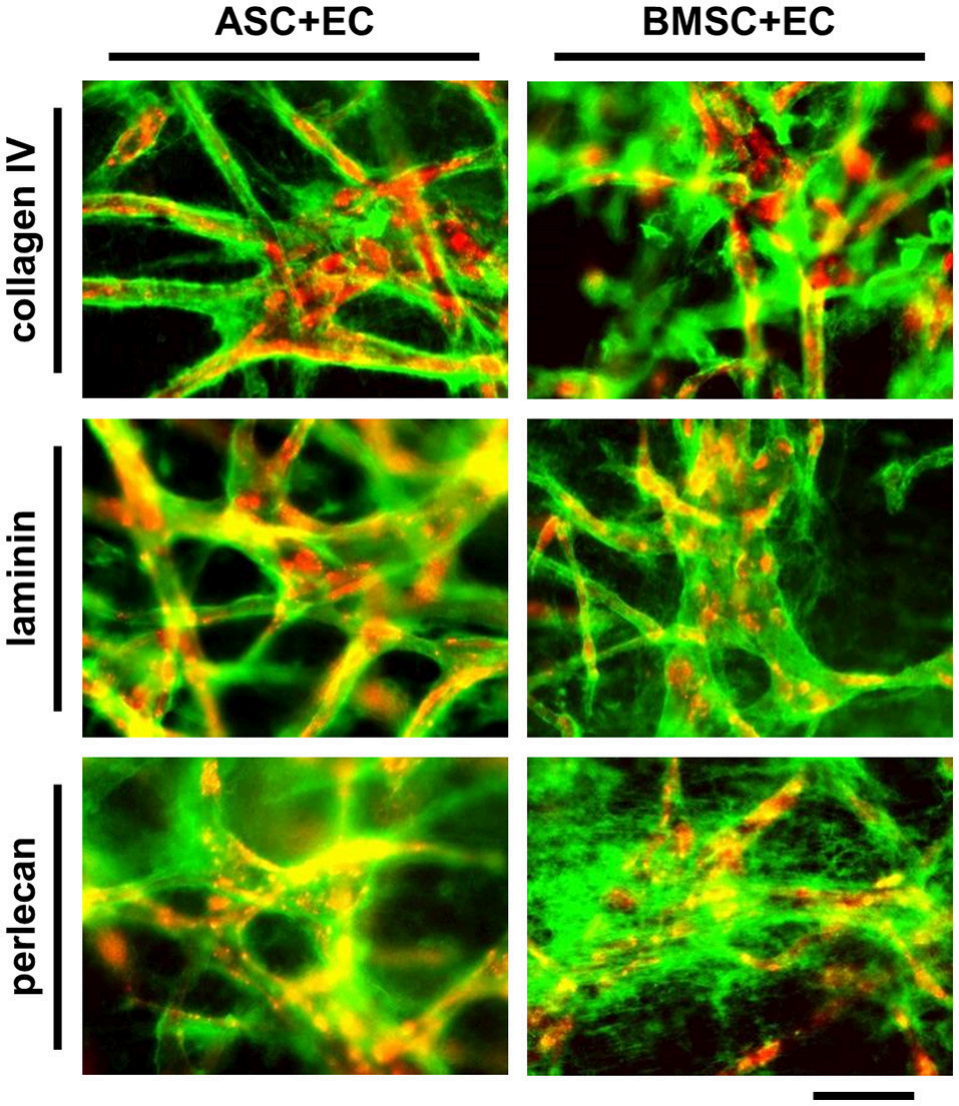
Vessel like structures in ASC + EC as well as BMSC + EC co-cultures are surrounded by basal lamina proteins after 1 week of culture. EC networks (red) are surrounded by collagen type IV, laminin and perlecan (green) after 1 week of culture. Scale bar: 300 μm.

Microvascular structures *in vivo* are surrounded by the basal lamina, which is composed of extracellular matrix (ECM) molecules including collagen type IV, laminin and perlecan (Laurie et al., 1982). The formation and localization of these molecules after 1 week of co-culture was assessed by immunostaining (Figure 3, EC in red, collagen IV, laminin and perlecan in green). Nearly all microvascular structures that formed within 1 week were surrounded by a layer of collagen type IV and laminin, while perlecan was located more diffusely throughout the matrix. Positive staining was detected in co-cultures of ASC+EC as well as in co-cultures of BMSC+EC without any noticeable differences between these two groups.

### Gene expression analysis reveals significant differences in ASC+EC co-cultures compared with BMSC+EC co-cultures

**Figure 4:**
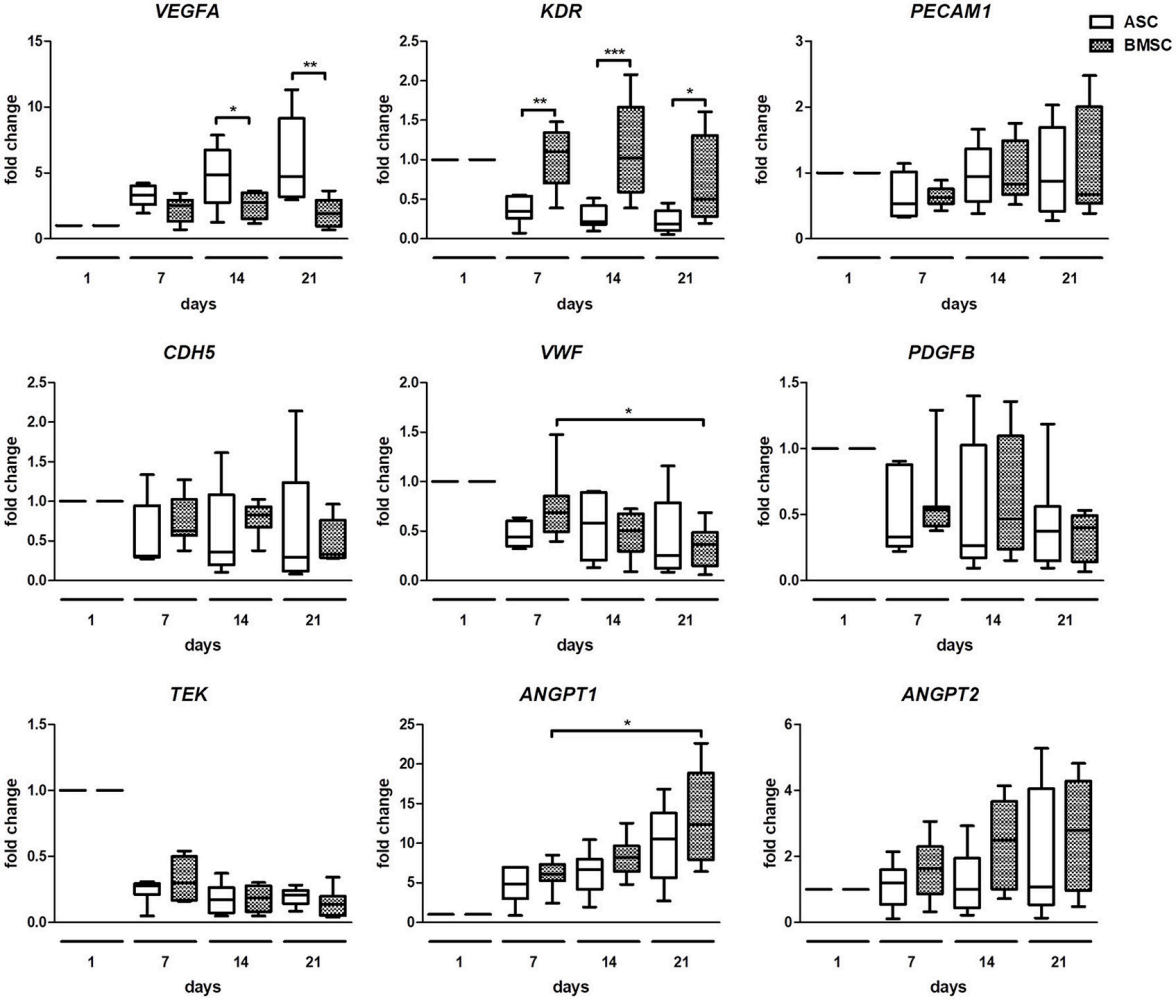
Gene expression analysis shows higher *VEGFA* expression in ASC + EC and higher *KDR* expression in BMSC + EC co-culture. *VEGFA* expression was significantly higher in ASC + EC on day 14 and day 21 while *KDR* expression was higher in BMSC+EC on day 7, day 14, and day 21. *PECAM1, CDH5, PDGFB*, and *ANGPT2* expression showed no significant changes. *ANGPT1* expression showed an increasing trend in both co-cultures, being significantly increased in BMSC + EC from day 7 to day 21. *n* = 7 or 8 from four biological replicates (each in technical duplicate), comparing two different donors of each cell type, *p < 0.05, **p < 0.01, ***p < 0.001, values are depicted as fold change and normalized to *GAPDH* and day 1.

To compare changes in angiogenic gene expression of both co-cultures, quantitative real time PCR was performed (Figure 4). The analysis revealed a significantly higher fold change of *VEGFA* in ASC+EC co-cultures compared to BMSC+EC co-cultures on day 14 and day 21. *KDR* increased significantly in BMSC+EC co-cultures on day 7, day 14, and day 21, compared to ASC+EC. Changes in the expression of *PECAM1, CDH5, PDGFB, TEK*, and *ANGPT2* were not significant. *ANGPT1* fold change increased significantly in BMSC+EC co-cultures from day 7 to day 21 and showed an increasing trend in ASC+EC co-cultures as well. Furthermore, the expression of *TEK* decreases in both co-cultures already after 1 week of culture.

## Discussion

The objective of this study was to investigate how MSC populations derived from different tissues influence vascular network formation, maturation and gene expression of EC. We show that while maturation of vascular networks in terms of coverage with NG2 expressing cells as well as basal lamina protein formation is similar in ASC+EC and BMSC+EC co-cultures, there are significant differences in vascular network density and gene expression. ASC as well as BMSC represent populations of post-natal tissue-specific stem cells with similar gene expression profiles (Lee et al., 2004) that are capable of secreting angiogenic factors like VEGF, FGF-2, and Ang-1 (Rohringer et al., 2014; Gallina, 2015). Both cell types are able to induce vascular network formation with a lower permeability compared to fibroblasts (Grainger and Putnam, 2011), which makes them an interesting cell source for tissue engineering purposes (Pill et al., 2015). While ASC and BMSC display similar characteristics, they differ e.g., in their differentiation (Xu et al., 2017) and proliferation potential (Kern et al., 2006), their capability for degrading the surrounding matrix during vessel formation (Ghajar et al., 2010; Kachgal et al., 2012; Holnthoner et al., 2015), and the potential of conditioned medium to induce outgrowth of EC (Verseijden et al., 2010b). Many studies report the use of co-culture models of MSC and EC in different 3D matrices to create microvessels, but only very few compare the potential of ASC and BMSC within the same setting (Pill et al., 2015). This study was conducted in order to reveal the differences in angiogenic potential of ASC compared to BMSC, as knowledge about such differences might be of importance for organotypic capillary formation. Understanding this heterogeneity of vascular beds is essential for developing novel diagnostics, vascular bed specific therapies and tissue engineering applications (Aird, 2007a,b; Augustin and Koh, 2017).

To investigate the differences in angiogenic potential of ASC and BMSC, co-cultures of ASC+EC and BMSC+EC were seeded in fibrin matrices, which has previously been established for EC co-cultures with ASC (Rohringer et al., 2014). We have shown previously, that ASC+EC co-cultures need not only growth factors added to the media, but also the direct cell-cell contact. Omitting either cells or factors will lead to a regression of the vascular network (Mühleder et al., 2018). Our results show that this model is also suitable for co-cultures with BMSC to induce network formation. To quantify changes in vascular network density, the parameters total tubule length, mean tubule length, number of junctions and number of tubules were evaluated. These parameters have not been compared between ASC+EC and BMSC+EC co-cultures before and confirmed observations in fluorescence images that a denser network with more and shorter tubules developed in co-cultures with ASC within 3 weeks than in co-cultures with BMSC.

Another important aspect of EC-MSC co-cultures is to what extent MSC differentiate toward a mural cell phenotype [pericyte (Guimarães-Camboa et al., 2017) or smooth muscle cell]. BMSC as well as ASC have been reported to migrate and localize in close proximity to vessels formed by EC, indicating a stabilizing role for these cells. Furthermore, MSC have been reported to express various pericyte markers including α-smooth muscle actin (α-SMA) (Merfeld-Clauss et al., 2010, 2014; Verseijden et al., 2010b; Carrion et al., 2013; Duttenhoefer et al., 2013), NG2 (Duttenhoefer et al., 2013; Rohringer et al., 2014; Eglinger et al., 2017), and CD146 (Sacchetti et al., 2007, 2016; Duttenhoefer et al., 2013). The presence of these markers indicates that MSC are not only involved in the initiation of vessel formation but also in maturation, as native vessels *in vivo* are surrounded by stabilizing structures composed of cells (pericytes or mural cells) as wells as basal lamina proteins. The generated networks in ASC+EC and BMSC+EC co-cultures presented here were covered with NG2 expressing cells after 3 weeks of culture, indicating formation of stabilized vasculature.

Next to the differentiation of MSC toward perivascular cells, the secretion and localization of proteins involved in basal lamina formation were investigated (Laurie et al., 1982). Microvascular matrix proteins collagen type IV as well as laminin and perlecan were produced by both co-cultures over the course of 1 week. While collagen type IV and laminin were located around vessel-like structures, perlecan did not accumulate at a specific site. There were no differences detected between both co-cultures. These data are in accordance with observations in ASC-EC co-cultures in which the deposition of collagen type IV and laminin was investigated next to perlecan and fibronectin (Merfeld-Clauss et al., 2010).

Differences between ASC+EC and BMSC+EC co-cultures were found in the fold change of angiogenesis related gene expression. While expression of *VEGFA*, a gene most crucial for angiogenesis (Ferrara, 2009), was significantly increased in ASC+EC co-cultures compared to BMSC+EC co-cultures on day 14 and day 21, *KDR* was significantly increased in BMSC+EC co-cultures at all investigated time points. The expression of *PECAM1, CDH5, VWF, PDGFB*, however, did not change in both co-cultures at all-time points. *TEK* expression was decreased (but not significant) in both co-cultures after the first week of culture and remained downregulated in the following weeks. In contrast, expression of *ANGPT1*, which is important for vascular remodeling, was significantly increased in BMSC+EC between day 7 and day 21, a trend that was also observed in ASC+EC. On the other hand, expression of *ANGPT2*, which has destabilizing functions in the microvasculature, showed no significant changes. These data indicate a quiescent and mature state of EC within microvessels, which is in accordance with previous findings of other groups (Saharinen et al., 2008; Pedersen et al., 2014).

Limitations of our study include that we cannot provide clear evidence which MSC type might be more “favorable” for specific vascular tissue engineering applications. However, rather than giving a recommendation on a specific cell type, we suggest to highlight that each MSC population has specific characteristics that have to be kept in mind when using them in different settings. Furthermore, there is a difficulty in distinguishing whether effects like the up-regulation of VEGF derives from ASC/BMSC and EC or from one cell type alone. It is challenging to assess each cell type individually in co-culture, and therefore, whether the number of cells and the ratios between the two cell types change. Another limitation of this study is the use of HUVEC. While this cell type is still used as the “golden standard” in vascular biology and tissue engineering, it is not present in the adult human body and therefore does not represent tissue-specific vasculature (Baiguera and Ribatti, 2013; Attalla et al., 2016). Similar to the shown differences in MSC sources, other EC harvested from distinct vascular beds might yield more insight in organotypic differences (Baiguera and Ribatti, 2013; Augustin and Koh, 2017). Additionally, the use of different expansion media for ASC and BMSC influences the behavior of these cell types and might also influence their potential to induce the formation of vascular structures in EC. However, we chose the most suitable media composition for expansion of each individual cell type, as ASC as well as BMSC have different needs in terms of media supplements (Suga et al., 2007). Choosing one media for expansion might have favored one cell type in advance.

Significant differences in network density and progression of network formation between ASC+EC and BMSC+EC co-cultures as well as higher expression of *VEGFA* hint toward a stronger angiogenic potential of ASC. Our study confirms the suitability of MSC populations from distinct tissues and donors to establish microvessel formation and maturation *in vitro* and highlights the importance of cell origin for relevant organotypic investigations. Although the two MSC types display similar effects on vascularization in co-culture with ECs, we suggest considering their differences in vascular tissue engineering applications.

## Author contributions

WH, SH, and KP contributed to experimental planning. KP performed experiments of the main manuscript and drafted the manuscript. JM isolated and characterized BMSC. SM isolated EC and performed retroviral infections. MP performed flow cytometric analysis. SR and EP isolated and characterized ASC. HR, SH, and WH edited the manuscript. All authors reviewed the manuscript.

### Conflict of interest statement

The authors declare that the research was conducted in the absence of any commercial or financial relationships that could be construed as a potential conflict of interest.
