# Supplementary material for: Microvascular Networks From Endothelial Cells and Mesenchymal Stromal Cells From Adipose Tissue and Bone Marrow: A Comparison
Source: Front Bioeng Biotechnol. 2018 Oct 25;6:156. doi: 10.3389/fbioe.2018.00156 (PMC6209673; doi:10.3389/fbioe.2018.00156)
Supplement: Supplementary file 1 [file Table_1.DOCX]

*Supplementary Material*

Microvascular networks from endothelial cells and mesenchymal stromal cells from adipose tissue and bone marrow: a comparison

**Karoline Pill^1,2^, Johanna Melke^3^, Severin Mühleder^1,2^, Marianne Pultar^1,2^, Sabrina Rohringer^4^, Eleni Priglinger^1,2^, Heinz Redl^1,2^, Sandra Hofmann^3^, Wolfgang Holnthoner^1,2^***

^1^ Ludwig Boltzmann Institute for Experimental and Clinical Traumatology, Vienna, Austria

^2^ Austrian Cluster for Tissue Regeneration, Vienna, Austria

^3^ Orthopaedic Biomechanics, Department of Biomedical Engineering, Eindhoven University of Technology, The Netherlands and Institute for Complex Molecular Systems, Eindhoven University of Technology, Eindhoven, The Netherlands

^4^ Department of Biomedical Research, Medical University of Vienna, Vienna, Austria

*** Correspondence:**

Dr. Wolfgang Holnthoner

wolfgang.holnthoner@trauma.lbg.ac.at

**S1 Figure: ASC as well as BMSC differentiate towards an adipogenic, osteogenic and chondrogenic phenotype and express typical MSC marker expression.** (a) Representative images are shown of differentiation in three lineages are shown. (b) ASC as well as BMSC are positive for CD73, CD90 and CD105 and negative for CD31, CD14 and CD45. Scale bar: 300 µm

**S2 Figure: EC cultured with ASC or BMSC form microvessels with lumen.** EC networks (green) show lumen (red arrows) in different sections derived by z-stack imaging, whether they are cultures with ASC (a) or BMSC (b). Scale bar: 50 µm

**Supplementary Methods**

## Adipogenic differentiation and detection

5∙10^3^ ASC or BMSC were seeded into wells of a 24 well plate and cultured in the corresponding growth media for three days. Media was then exchanged to either control medium (DMEM HG, 10% FCS [Sigma-Aldrich], 1% Penicillin/Streptomycin [Sigma-Aldrich], 1% L-glutamine [Sigma-Aldrich]) or differentiation medium (DMEM HG, 10% FCS, 1% Penicillin/Streptomycin, 1% L-glutamine, 1 µM dexamethasone [Sigma-Aldrich], 0.5 µM 3-isobutyl-1-methylxanthine [Serva], 0.5 mM hydrocortisone [Sigma-Aldrich] and 60 µM indomethacin [Sigma-Aldrich]). Media were sterile filtered prior to usage. ASC and BMSC were cultured for 14 days, during which media was exchanged every 2-3 days.

After 14 days, medium was discarded and the cells washed three times with PBS. Subsequently, 4% formaldehyde solution was added for 30 min at RT. The cells were then washed three times with distilled water. Afterwards, 70% ethanol was added for 2 min at RT prior to adding Oil Red O working solution (3 mg/ml Oil Red O dissolved in isopropanol, filtered, diluted with distilled water 1:0.875 and filtered again) for 10 min at RT. Samples were washed three times with distilled water prior to imaging (Zeiss Primovert Microscope with ICc5 axiocam, Zeiss).

**Osteogenic differentiation and detection**

For osteogenic differentiation, ASC after one week in culture were seeded at a density of 2x10^3^ cells per well in a 24-well plate in EGM-2 and incubated overnight. The next day, medium was changed to osteogenic differentiation medium DMEM-low glucose (Lonza) containing 10% FCS, 2 mM L-glutamine (PAA), 100 U/ml Penicillin/Streptomycin (Lonza), 10 nM dexamethasone, 150 µM ascorbat-2-phosphate (Sigma), 10 mM β-glycerophosphate (StemCell Technologies) and 10 nM dihydroxy-vitamin D3 (Sigma) or control medium consisting of DMEM:F12 / L-glutamine with 10% FCS and 100 U/ml Penicillin/Streptomycin. Medium was changed every 3 to 4 days. After 21 days, osteogenic differentiation was analyzed with Alizarin Red staining. Cells were fixed for 1 hour with 70% ethanol at -20°C and stained with Alizarin Red solution (Merck) for 15 minutes.

To assess the potential of hBMSCs for osteogenic differentiation, cells were trypsinized and resuspended in either control medium (DMEM supplemented with 10% FBS, Penicillin/Streptomycin and Fungizone) or osteogenic medium (control medium supplemented with 50 µg/ml ascorbic acid-2-phosphate, 100 mM dexamethasone, 10 mM β-glycerolphosphate). Aliquots of 1 ml containing 10^5^ cells were seeded in 24-well plates. Medium was changed every 2–3 days. After 3 weeks of culture, cells were washed twice in PBS, fixed in 10% neutral buffered formalin and stained with Alizarin Red.

**Chondrogenic differentiation and detection**

For chondrogenic differentiation in 3D micromass pellet cultures, 3x10^5^ ASC after one week in culture were centrifuged in chondrogenic differentiation medium (hMSC Chondro BulletKit [Lonza] containing 10 ng/ml BMP-6 [R&D] and 10 ng/ml TGF-ß3 [Lonza]) in screw cap micro tubes. The tubes were placed in an incubator with slightly open caps for gas exchange. After 2 days, the pellets were transferred to 96-well U-bottom plates (Greiner) with fresh medium. Medium was changed every 2 to 3 days. After 35 days of differentiation, micromass pellets were fixed in 4% phosphate-buffered formalin overnight for histological analysis with Alcian Blue. The next day the pellets were washed in 1x PBS and dehydrated in increasing concentrations of alcohol. After rinsing the pellets in xylol and infiltration with paraffin, deparaffinized sections were stained with Alcian blue for 30 minutes and counterstained for 2 minutes with hematoxylin.

BMSCs were trypsinized and resuspended in either control medium (DMEM supplemented with 10% FBS, Penicillin/Streptomycin and Fungizone), or chondrogenic medium (control medium supplemented with 0.1 mM nonessential amino acids, 50 µg/ml ascorbic acid-2-phosphate, 10 nM dexamethasone, 5 µg/ml insulin, 5 ng/ml TGF-β1). Aliquots containing 2×10^5^ cells were centrifuged for to form pellets. Medium was changed every 2–3 days. After 4 weeks of culture, pellets were washed twice in PBS, fixed in 10% neutral buffered formalin, embedded in paraffin and sectioned (5 µm thick). Sections were stained for glycosaminoglycans with Alcian blue.

## Flow cytometry analysis

MSCs were washed once with PBS and detached with accutase (Sigma-Aldrich). They were subsequently centrifuged, resuspended in 800 µl PBS containing 1% bovine serum albumin. 2 µL of the fluorescent-conjugated antibody were added to 100 µL of the cell suspension. ASC were stained using IgG-FITC (BioLegend), IgG-PE (BD Bioscience), CD73-PE (BD Bioscience), CD90-PE (BD Bioscience), CD105-FITC(BD Bioscience), CD31-PE (BD Bioscience), CD14-FITC (ImmunoTools) and CD45-FITC (ImmunoTools). For the staining of BMSC the same antibodies were used. Aside from that a PE labeled CD105 antibody (Abcam) was added. After incubation for 30 min on ice in the dark cells were washed twice with PBS containing 1% bovine serum albumin. Cells were resuspended in 300 µL PBS containing 1% bovine serum albumin prior to flow cytometry analysis on a FACS Canto II flow cytometer (BD Biosciences). Data were analyzed using FlowJo software (TreeStar).
