# Supplementary figures and images for: Microvascular Networks From Endothelial Cells and Mesenchymal Stromal Cells From Adipose Tissue and Bone Marrow: A Comparison
Source: Front Bioeng Biotechnol. 2018 Oct 25;6:156. doi: 10.3389/fbioe.2018.00156 (PMC6209673; doi:10.3389/fbioe.2018.00156)

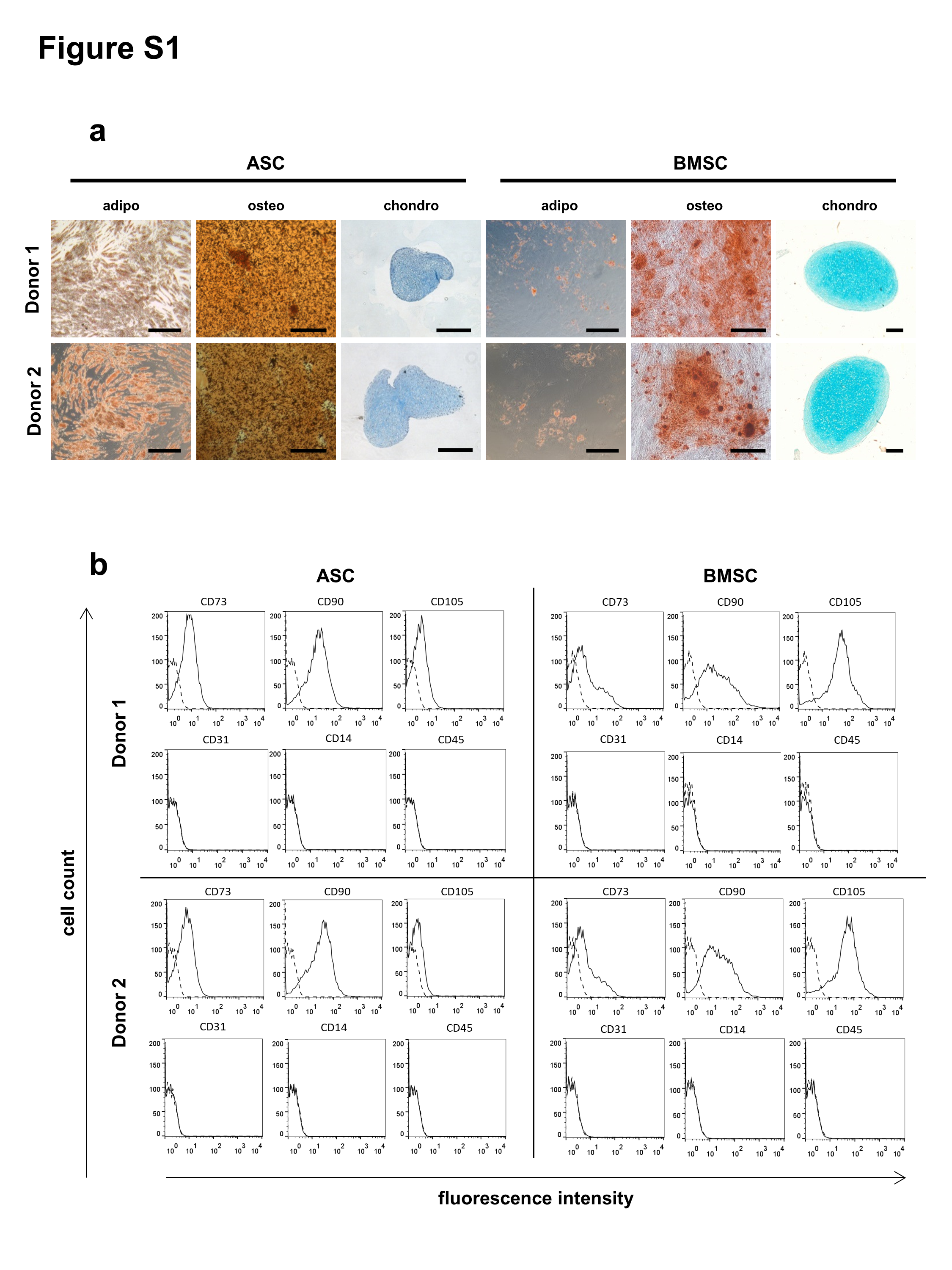

Supplement: Supplementary file 2 [file Image_1.TIF]

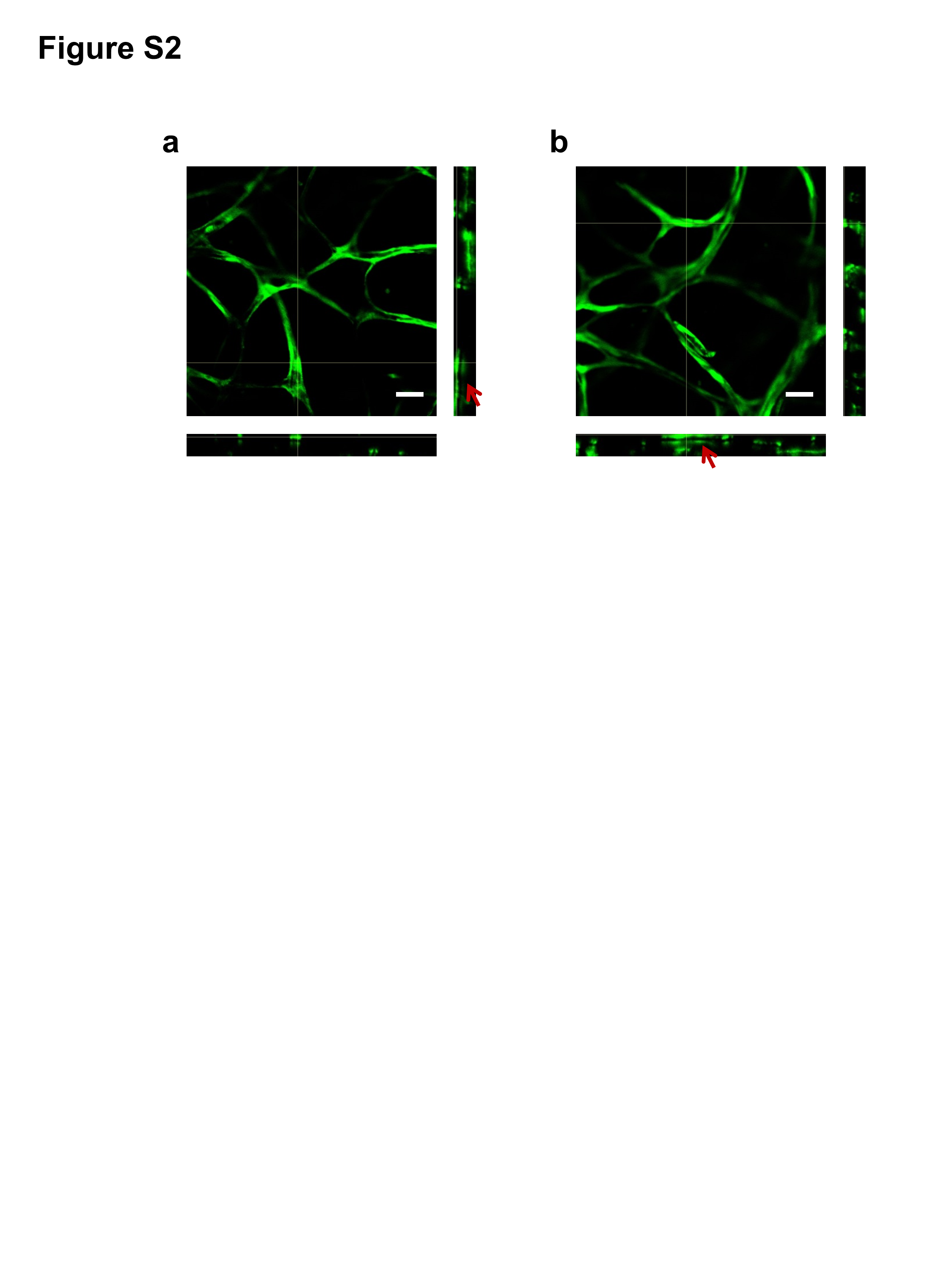

Supplement: Supplementary file 3 [file Image_2.TIF]
